# Identification of a Diagnostic Signature and Immune Cell Infiltration Characteristics in Keloids

**DOI:** 10.3389/fmolb.2022.879461

**Published:** 2022-05-20

**Authors:** Yijun Xia, Youbin Wang, Yingjie Xiao, Mengjie Shan, Yan Hao, Lingyun Zhang

**Affiliations:** ^1^ Department of Plastic Surgery, Peking Union Medical College Hospital, Chinese Academy of Medical Sciences, Peking Union Medical College, Beijing, China; ^2^ Department of Plastic Surgery, Peking Union Medical College Hospital, Beijing, China; ^3^ Department of Cardiothoracic Surgery, Second Affiliated Hospital, School of Medicine, Zhejiang University, Hangzhou, China; ^4^ Department of Plastic Surgery, Heze Municipal Hospital, Heze, China

**Keywords:** keloid, diagnostic signature, immune cell infiltration, ssGSEA, TGM2

## Abstract

**Background:** Keloid disorder is a recurrent fibroproliferative cutaneous tumor. Due to the lack of early identification of keloid patients before the formation of keloids, it is impossible to carry out pre-traumatic intervention and prevention for these patients. This led us to identify and determine signatures with diagnostic significance for keloids.

**Methods:** Public series of matrix files were downloaded from the Gene Expression Omnibus database. Differentially expressed genes (DEGs) were calculated from expression profiling data, and the diagnostic signature was identified by constructing a protein-protein interaction (PPI) network. The diagnostic efficacy of the screened signature was assessed by employing receiver operating characteristic (ROC) curves. Furthermore, we calculated the proportion of different immune cells in the gene expression matrix microenvironment by the “ssGSEA” algorithm, and assessed the difference in immune cell abundance between keloids and control groups and the relationship between the signature and immune cell infiltration. Clinical keloid and normal skin tissues were collected, and the expression of the screened diagnostic signature was validated by RT-qPCR and immunohistochemical assay.

**Results:** By screening the key genes in PPI, TGM2 was recognized and validated as a diagnostic signature and the infiltrating abundance of 10 immune cells was significantly correlated with TGM2 expression. Gene ontology enrichment analysis demonstrated that TGM2 and molecules interacting with it were mainly enriched in processes involving wound healing and collagen fiber organization. TGM2 correlated positively with HIF-1A (R = 0.82, *p*-value = 1.4e-05), IL6 (R = 0.62, *p*-value = 0.0053), and FN1 (R = 0.66, *p*-value = 0.0019). Besides, TGM2 was significantly upregulated in clinical keloid samples compared to normal skin tissues.

**Conclusion:** TGM2 may serve as an auxiliary diagnostic indicator for keloids. However, the role of TGM2 in keloids has not been adequately reported in the current literature, which may provide a new direction for molecular studies of keloids.

## Introduction

Keloid disorder is a manifestation of excessive healing after skin injury, characterized by abnormal proliferation of fibroblasts and deposition of extracellular matrix, and is common in plastic surgery (Grabowski et al., 2020). Keloids are more frequent on some exposed parts of the skin with pain and itching, and improper care can induce infection and septicemia, causing considerable physical and psychological stress to the patient (Lee et al., 2019). However, the current diagnoses of keloids primarily rely on the clinical manifestations and associated scar scales. Patients with a tendency to develop keloids can be diagnosed only after the formation of keloids after surgery or trauma, which notably impacts the risks and efficacy of open surgery. Therefore, it is urgent to search for reliable diagnostic indicators for early screening and prevention of keloids to avoid unnecessary surgical stimulations.

Gene sequencing is able to determine the exact relationships among some gene loci and diseases, which has guiding significance for clinical diagnosis and treatment (Horgan et al., 2017). The advances in bioinformatics provide the ability to assist the secondary utilization of existing sequencing data, fully mine the information that is provided by the sequencing data, and identify the biomarkers for a variety of diseases (Min et al., 2017). As a highly recurrent fibrous proliferative tumor, in the absence of satisfactory drug and surgical treatment, the identification of early indicative biomarkers in keloid is of key significance for effective prevention, risk reduction and development of targeted therapy. At present, the identification of keloid biomarkers mostly relies on the reference of other fibrosis diseases, such as studying the role of scleroderma biomolecules in keloids. However, the efficiency of this method is relatively low, and it is prone to omit the specific markers of keloids. We thus utilized gene sequencing datasets and bioinformatics methods to filter keloid markers in the expectation of more efficient identification of key signatures of keloids.

In this study, public series of matrix files for keloids were downloaded from the Gene Expression Omnibus (GEO) database. Pivotal molecules were screened based on the PPI network and the diagnostic efficacy of the screening signature was validated by applying receiver operating characteristic (ROC) curves, and the association of the filtered signature with immune cell infiltration was evaluated to gain more insight into the molecular immune mechanisms involved in keloids.

## Materials and Methods

### Data Downloading and Processing

The keloid matrix files that are freely available in the Gene Expression Omnibus (GEO) database were filtered with the organization as “Homo sapiens” and study type as “Expression profiling by array” or “Expression profiling by high throughput sequencing” as the restriction conditions (Wang et al., 2019). We filtered three different microarray and RNA-seq datasets with accession numbers GSE7890, GSE145725 and GSE117887 (Table 1). The “GEOquery” package of R software (version 3.6.3, R Foundation) was applied to download the series of matrix and probe annotation files (Davis and Meltzer 2007) and the annotation information was extracted through the “affy” package to complete the conversion of probe names to gene symbols (Gautier et al., 2004). The RMA algorithm was employed to perform background corrections to exclude the probe signals from being altered by some nonspecific factors.

**TABLE 1:** Overview of the datasets used in this study.

Dataset	Platform	Study Type	Sample	Country
GSE7890	Affymetrix Human Genome U133 Plus 2.0 Array	Expression profiling by array	GSM194109	United States
			GSM194110	
			GSM194111	
			GSM194112	
			GSM194113	
			GSM194118	
			GSM194119	
			GSM194120	
			GSM194121	
			GSM194122	
GSE145725	GeneChip^®^ PrimeView™ Human Gene Expression Array (with External spike-in RNAs)	Expression profiling by array	GSM4331585	United States
			GSM4331586	
			GSM4331587	
			GSM4331588	
			GSM4331589	
			GSM4331590	
			GSM4331591	
			GSM4331592	
			GSM4331593	
			GSM4331594	
			GSM4331595	
			GSM4331596	
			GSM4331597	
			GSM4331598	
			GSM4331599	
			GSM4331600	
			GSM4331601	
			GSM4331602	
			GSM4331603	
GSE117887	Illumina HiSeq 3,000 (Homo sapiens)	Expression profiling by high throughput sequencing	GSM3314472	Australia
			GSM3314473	
			GSM3314474	
			GSM3314475	
			GSM3314476	
			GSM3314477	
			GSM3314484	
			GSM3314485	
			GSM3314486	
			GSM3314487	
			GSM3314488	
			GSM3314489	

### Differentially Expressed Genes Screening

RNA-seq datasets and microarray based on gene expression analysis are sensitive methods to identify possible genes and molecular pathways. Since the data came from different sources, we preprocessed our data through Z-score transformation and adopted log_2_ transformation to alleviate the influence of outliers. The algorithm of the “limma” package (http://www.bioconductor.org/) was adopted to extract the differential genes from the expression profiles (Ritchie et al., 2015) and P.value <0.05 and |log fold change (FC)| >2 were considered to be the threshold points to filter the genes that were significantly differentially expressed in keloids (Ito and Murphy 2013).

### Protein–Protein Interaction Analysis

In the STRING (http://string-db.org/) database, the “Multiple Proteins” model was used to construct a protein–protein interaction (PPI) network of genes differentially expressed in both GSE7890 and GSE145725 to demonstrate the links between genes (Szklarczyk et al., 2017). The molecules of the protein network were ranked for connectivity in Cytoscape software and the highest ranked molecule was selected as the key molecule.

### Gene Functional Enrichment and Annotation

The GeneMANIA database was employed to construct a network of interacting proteins with the screened signature (Warde-Farley et al., 2010). To provide additional insights into the biological significance of gene expression differences and the mechanisms that contribute to keloid traits, we performed gene ontology (GO) enrichment analysis of the screened signature and its interacting genes to predict possible involvements in cellular components, molecular functions and biological processes.

### Correlation Analysis

To investigate the possible molecular mechanism of the screened signature in keloid, we conducted correlation analysis between the screened signature and the widely studied molecules and recognized signaling pathways in keloids. Gene sets involved in extracellular matrix (ECM) formation, collagen formation, hypoxia and epithelial mesenchymal transition (EMT), and the TGF-beta signaling pathway were obtained in the molecular signatures database (MSigDB) (Liberzon et al., 2015), and correlations between these molecules and the screened signature were visualized by radar plots and scatter plots.

### Immune Cell Infiltration

Keloids consist not only of abnormally proliferating fibroblasts but also consist of different cell types, including matrix cells and immune cells, which form the microenvironment of keloids. This led us to compute the proportion of different immune cells in the gene expression matrix microenvironment through the “ssGSEA” algorithm (Subramanian et al., 2005; Bindea et al., 2013) and to evaluate the difference in immune cell abundance between keloid and normal skin and the relationship between the screened signature and immune cell infiltration.

### Validation of the Diagnostic Signature

The difference in the expression of the filtered diagnostic signature between keloid and non-keloid was verified in GSE7890, GSE145725 and GSE117887. The receiver operating characteristic (ROC) curve was poltted with the R software “pROC” package (Mandrekar 2010) and the area under the curve (AUC) was calculated to reflect the predictive abilities of the screened signature in GSE7890, GSE145725 and GSE117887 (Robin et al., 2011).

### Ribonucleic Acid Extraction and Reverse Transcription -Quantitative Polymerase Chain Reaction Technology

The study protocol was approved by the Bioethical Committee of Peking Union Medical College Hospital. Fifteen keloid samples and five normal skin samples were randomly collected from Department of Plastic Surgery at Peking Union Medical College Hospital. Samples were frozen immediately after leaving the body and kept at -80°C. Total RNA was extracted from samples by a Tissue RNA Kit (G3013, Servicebio). RNA was reverse-transcribed into cDNA using Servicebio^®^RT First Strand cDNA Synthesis Kit (G3330, Servicebio). The sequences of primer pairs used in the study are shown in Table 2. Real-time qPCR was performed using the SYBR Green qPCR Master Mix (2X) (G3320, Servicebio). Real-time qPCR cycle parameters included initial denaturation at 95°C for 10 min followed by 40 cycles involving denaturation at 95°C for 15 s, annealing at 60°C for 30 s, and extension at 60°C for 30 s. Each sample was tested thrice within the same run. PCR data were normalized using the 2^−ΔΔCT^ method; Glyceraldehyde 3-phosphate dehydrogenase (GAPDH) was used as internal reference for mRNA expression.

**TABLE 2:** Primers and their sequences for Quantitative RT-PCR analysis.

Gene Name	Sense Primers	Antisense Primers
TGM2	5′-CTC​ACC​CAG​CAG​GGC​TTT​AT-3′	5′-CCA​TCT​TCA​AAC​TGC​CCA​AAA-3′
GAPDH	5′-GGA​AGC​TTG​TCA​TCA​ATG​GAA​ATC-3′	5′-TGA​TGA​CCC​TTT​TGG​CTC​CC-3′

### Immunohistochemical Assay

Eight cases of formalin-fixed, paraffin-embedded keloid tissue and eight cases of normal skin tissue were collected. According to the streptavidin-biotin-peroxidase complex procedure, keloid tissue sections were dewaxed and rehydrated with xylene and different concentrations of ethanol, and the pretreatment of keloid sections was completed. The primary antibody working solution was incubated at 37°C overnight at 4°C. Biotin-labeled secondary antibodies were incubated at 37°C for 30 min. An appropriate amount of alkaline phosphatase-labeled streptavidin working solution was added and incubated at 37°C for 30 min. Sections were re-stained, dehydrated and sealed. TGM2 antibody (dilution = 1:250, ab109200, Abcam, Cambridge, United Kingdom) was used to detect TGM2 expression. Quantitative results were obtained using ImageJ (version 1.8.0) to calculate the integrated optical density (IOD) of positive markers.

### Statistical Analysis

All statistical analyses were performed in R software (version 4.1.2) with a two-tailed *p*-value < 0.05 as statistically significant. Pearson analysis was employed to assess correlations between gene expression values, and between gene expression and immune cell scores. Continuous variables between keloid and control groups were compared using the Mann-Whitney U-test. Receiver operating characteristic (ROC) curves were adopted to estimate the predictive value of the screened signature in keloid patients, and the value of the area under the curve (AUC) was taken to express the diagnostic efficacy of the screened signature.

## Results

### Data Processing

**FIGURE 1:**
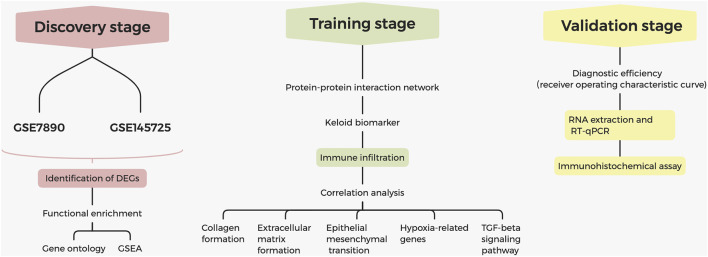
Flowchart for identification of diagnostic signatures in keloids.

**FIGURE 2:**
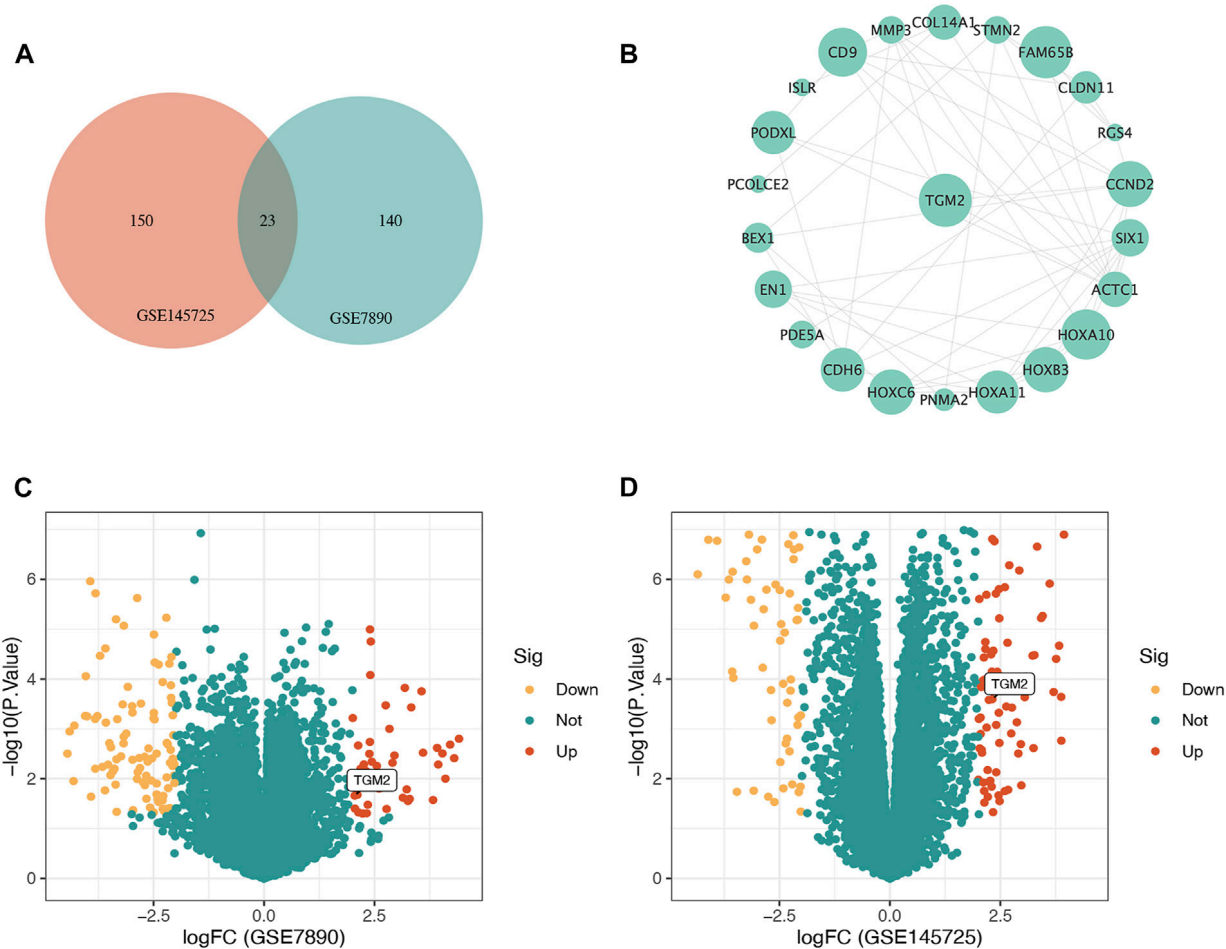
Illustration of the identification of the hub gene in keloid. **(A)** Venn diagram demonstrating the intersection of differentially expressed genes (DEGs) in GSE7890 and GSE145725. **(B)** Protein-protein interaction network constructed based on STRING database and Cytoscape software for DEGs shared by GSE7890 and GSE145725, and TGM2 with the highest connectivity was selected as the key gene. **(C)** Volcano map of DEGs of the GSE7890 dataset. **(D)** Volcano map of DEGs of the GSE145725 dataset. Red represents up-regulated genes, green represents non-significant genes, and yellow represents down-regulated genes.

The process of screening signatures is shown in Figure 1. We downloaded the gene expression microarray and RNA-seq from the GEO database (Table 1) and completed the annotation of gene symbols and clinical grouping information in the R software package. The DEGs in the GSE7890 and GSE145725 datasets were extracted by the " limma” package (Figure 2), and 23 intersecting genes were obtained as illustrated in the venn diagram.

### Protein-Protein Interaction Analysis

A protein-protein interaction network of genes differentially expressed in both GSE7890 and GSE145725 datasets was constructed in the STRING database and imported into Cytoscape software. The gene with the highest connectivity to neighboring genes was screened as the key gene, namely TGM2 (Figure 2B).

### Enrichment Analyses

**FIGURE 3:**
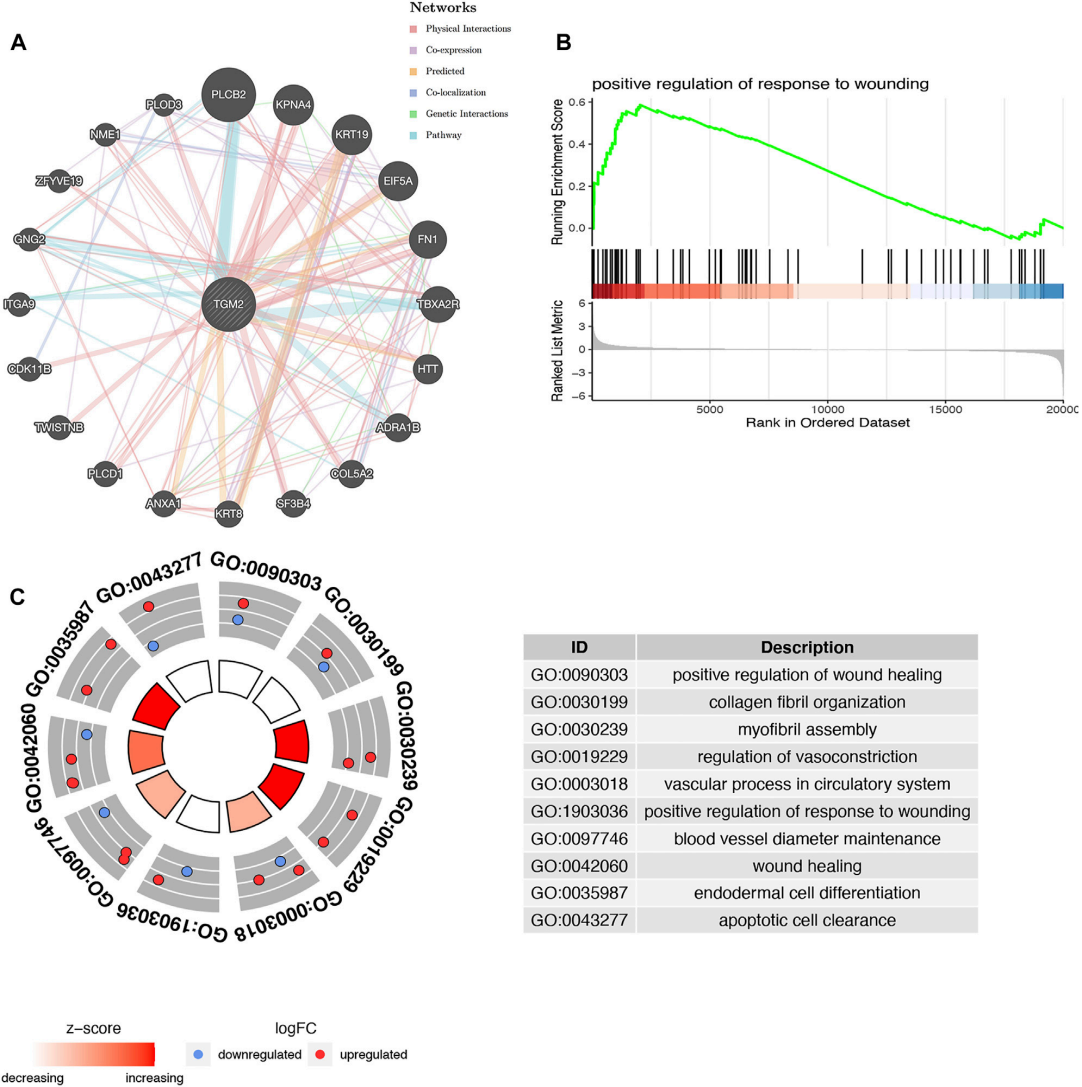
Functional prediction of TGM2. **(A)** Interaction network of TGM2 and its possible related genes was constructed in the GeneMANIA database. **(B)** Enrichment analyses of differentially expressed genes via gene set enrichment analysis. **(C)** Biological function prediction of TGM2 and its related genes based on gene ontology enrichment analysis.

A protein network interacting with TGM2 was achieved through bioinformatics approaches based on physical interaction, co-expression, co-localization, pathway, prediction and genetic interactions (Figure 3A). Based on the results of the enrichment scores, DEGs may be associated with the regulation of wounding (Figure 3B). GO enrichment analysis demonstrated that TGM2 and molecules interacting with it were mainly enriched in processes involving wound healing, collagen fiber organization, myofibril assembly, regulation of vasoconstriction, and blood vessel diameter maintenance (Figure 3C).

### Immune Cell Infiltration

**FIGURE 4:**
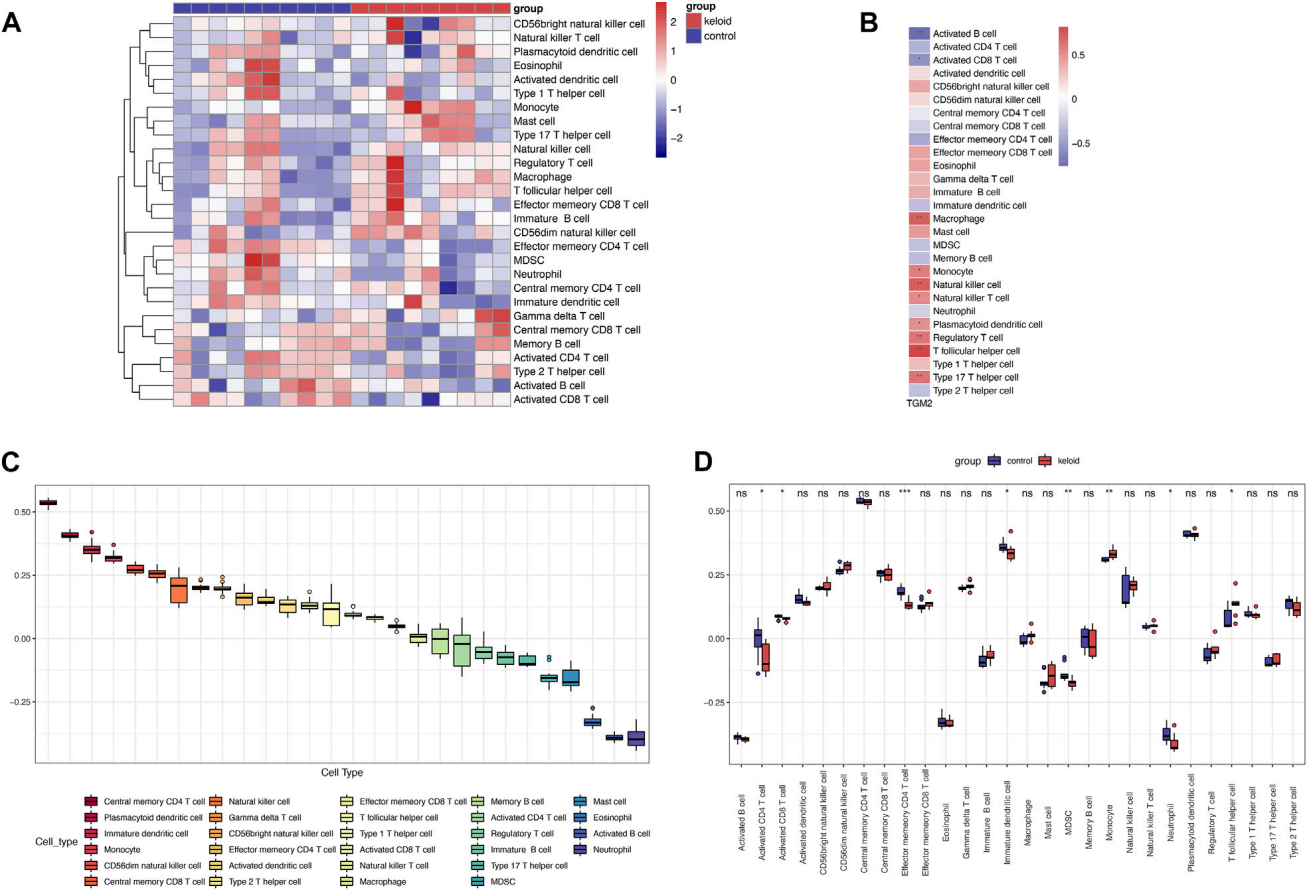
Evaluation and visualization of immune cell infiltration. **(A)** Heatmap of immune cells in keloid and control groups via single-sample gene set enrichment analysis in GSE145725 dataset (*n* = 19). **(B)** Correlation analysis of TGM2 expression with immune cell infiltration in the GSE145725 dataset. **(C)** Ranking of immune cell scores in the GSE145725 dataset. **(D)** Variation of immune cell scores between keloid and control groups in the GSE145725 dataset. *p < 0.05; **p < 0.01; ***p < 0.001; ****p < 0.0001. ns, not significant.

Single sample gene set enrichment analysis (ssGSEA) was performed to analyze the GSE145725 dataset and revealed the infiltration of 28 immune cell subpopulations in keloids (Figure 4), with central memory CD4 T cell scoring highest among the immune cells. The results identified significant differences in the proportion of eight immune cell types in keloid and non-keloid tissues, namely activated CD4 T cell, activated CD8 T cell, effector memory CD4 T cell, immature dendritic cell, MDSC, monocyte, neutrophil and T follicular helper cell. The infiltrating abundance of 10 immune cells was significantly correlated with TGM2 expression, among which activated B cell and activated CD8 T cell were negatively correlated with TGM2.

### Correlation Analysis

**FIGURE 5:**
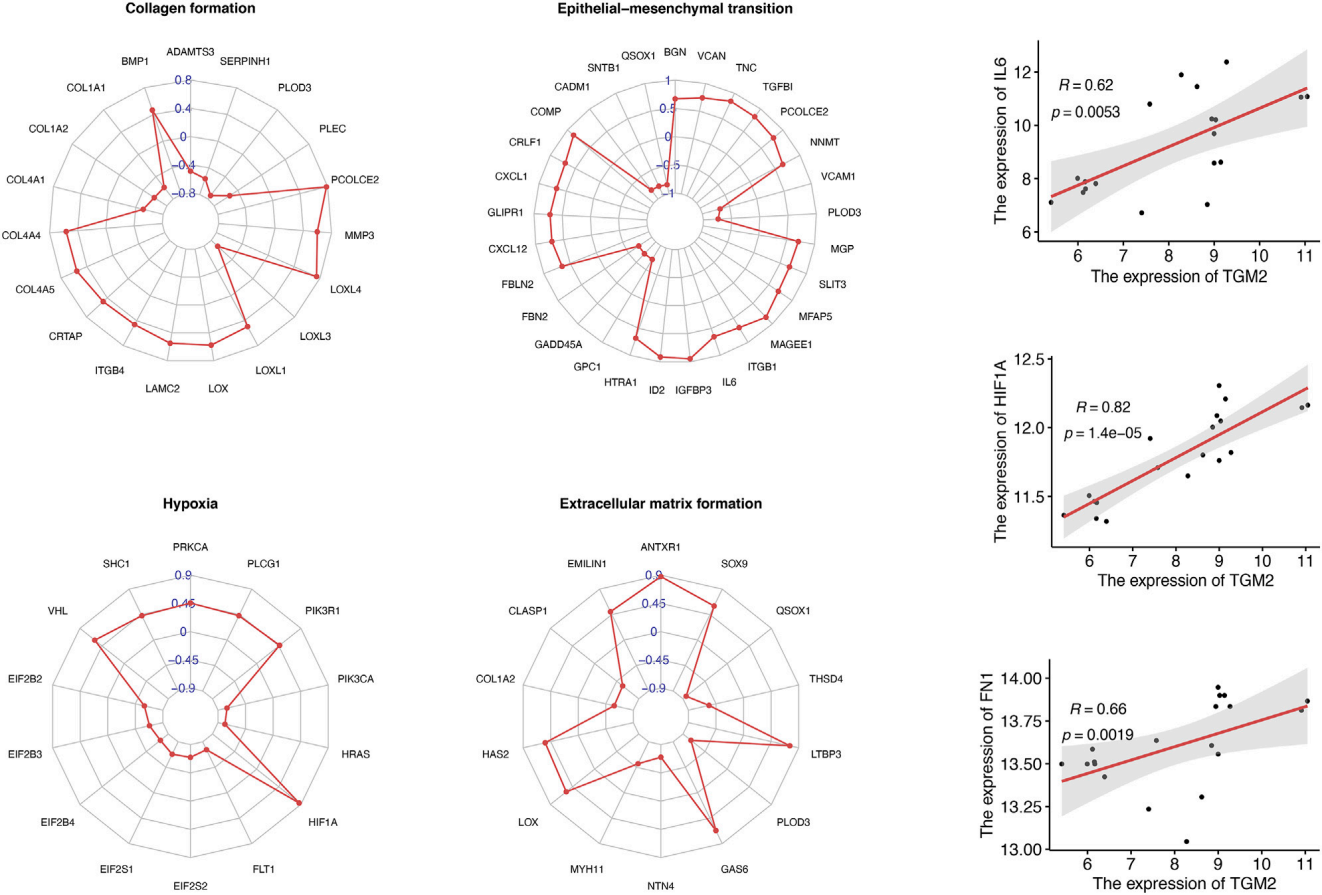
Correlation analysis of TGM2 and keloid-associated genes. Radar plots summarized the genes associated with TGM2 in the gene sets of collagen formation, epithelial mesenchymal transition, hypoxia and extracellular matrix formation. Correlation coefficients between TGM2 and keloid-associated genes were indicated in blue. Scatter plots demonstrated significant positive correlations between TGM2 and interleukin 6 (IL6), hypoxia inducible factor 1 subunit alpha (HIF1A), and fibronectin 1 (FN1).

**FIGURE 6:**
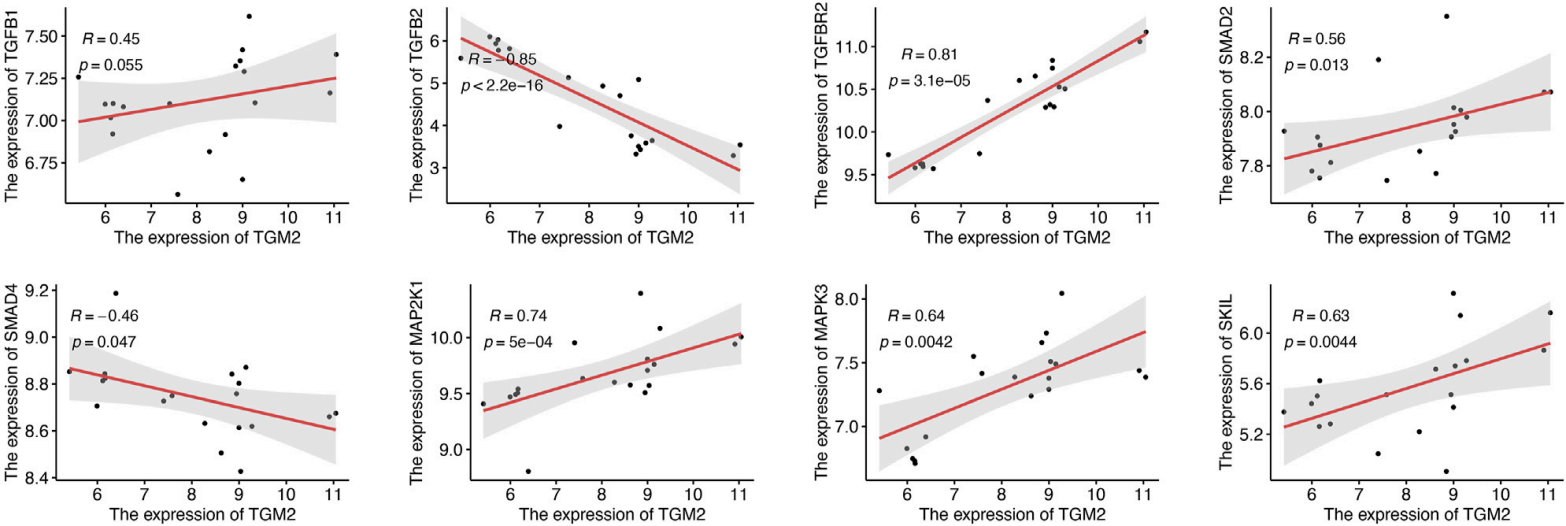
Correlation analysis of TGM2 and the TGF-beta signaling pathway in keloids. The scatter plots illustrated the molecules that were significantly correlated with TGM2. Due to the important role of TGF-β1 in the TGF-beta signaling pathway, the scatter plot of the correlation between TGF-β1 and TGM2 was retained.

To investigate the possible molecular mechanism of TGM2 in keloids, we selected key molecules involved in extracellular matrix formation, collagen formation, hypoxia and epithelial mesenchymal transition to conduct correlation analysis with TGM2 in the GSE145725 dataset (Figure 5). TGM2 correlated positively with HIF-1A (R = 0.82, *p*-value = 1.4e-05), IL6 (R = 0.62, *p*-value = 0.0053), and FN1 (R = 0.66, *p*-value = 0.0019). Subsequently, we analyzed the correlation between TGM2 and the TGF-beta signaling pathway in keloids (Figure 6). TGM2 was positively correlated with TGFBR2 (R = 0.81, *p*-value = 3.1e-05) and SMAD2 (R = 0.56, *p*-value = 0.013), and significantly negatively correlated with SMAD4 (R = -0.46, *p*-value = 0.047) and TGFB2 (R = -0.85, *p*-value < 2.2e-16).

### Validation of the Diagnostic Signature

**FIGURE 7:**
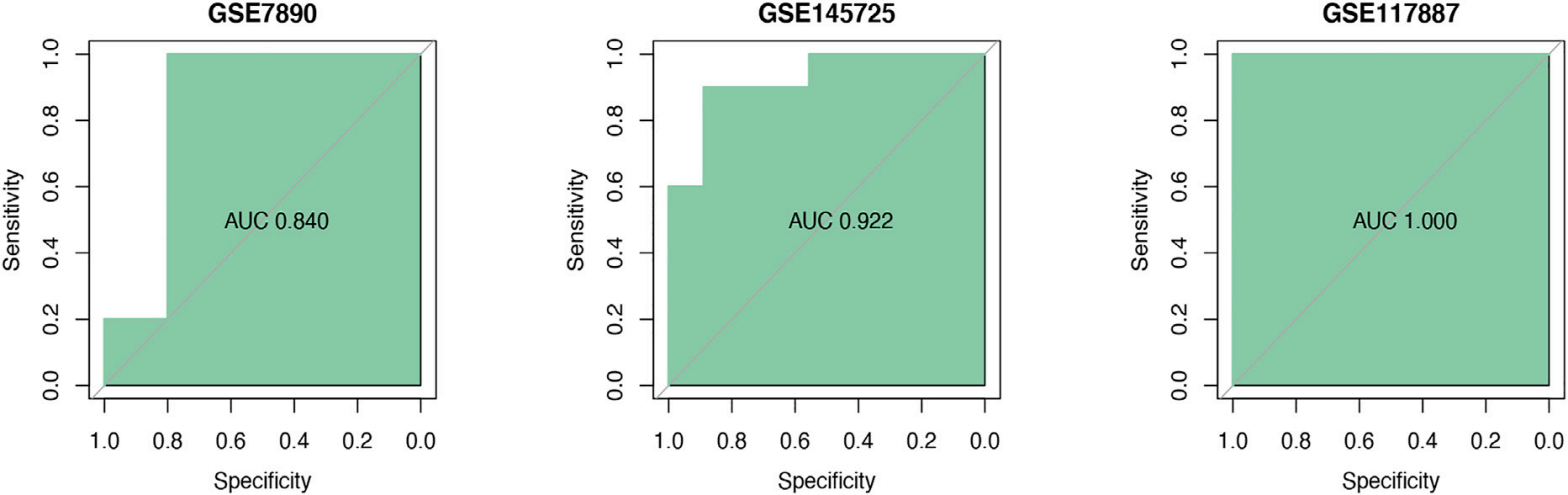
Verification of the diagnostic signature. The diagnostic efficacy of filtered TGM2 was verified by ROC curves in three datasets GSE7890 (*n* = 10), GSE145725 (*n* = 19), and GSE117887 (*n* = 12).

**FIGURE 8:**
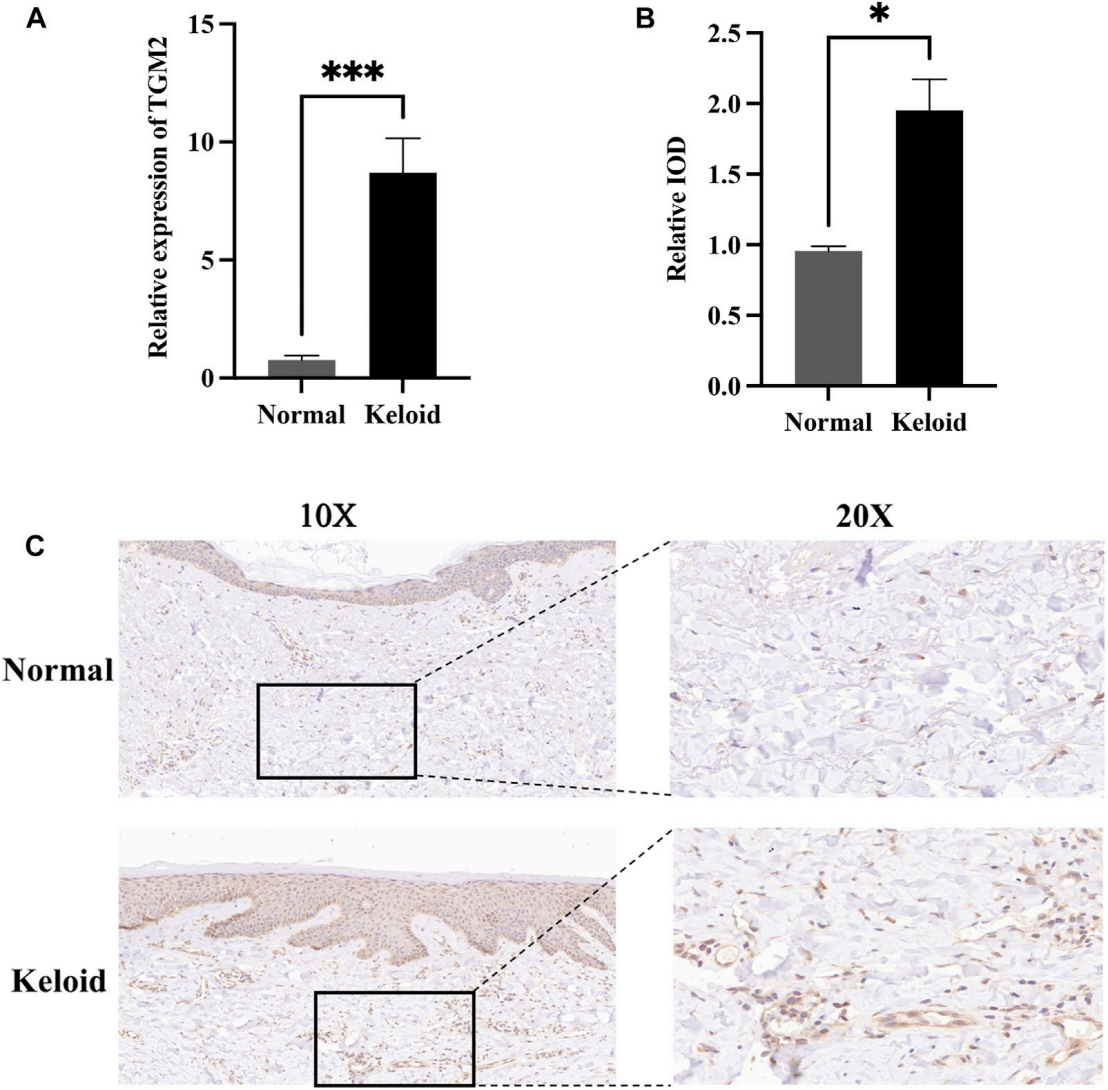
Expression of TGM2 in keloids and normal skin tissues. **(A)** The expression of TGM2 in keloid tissues confirmed by RT-qPCR. The expression of TGM2 mRNA in keloids was significantly higher than that in normal skin tissues (P.value = 0.0003). **(B)** The relative IOD (integrated optical density) value of TGM2 in keloids and normal skin tissues. **(C)** Immunohistochemical staining of TGM2 in keloids and normal skin tissues.

ROC curves indicated that the AUC values of TGM2 in GSE7890, GSE145725 and GSE117887 were 0.840, 0.922 and 1.000, respectively, indicating that TGM2 has a robust performance in the diagnosis of keloids (Figure 7). In addition, we detected TGM2 expression in clinical keloid tissues via RT-qPCR and IHC (Figure 8). The results revealed that the expression of TGM2 was significantly increased in keloid tissues compared with the corresponding normal skin tissues.

## Discussion

Keloid disorder is very common in the clinical work of plastic surgery, yet due to the lack of clear pathogenesis and effective treatment measures, it is a refractory disease (Lee and Jang 2018). Pathologically, keloids have tumor-like growth characteristics, which chiefly manifest as excessive fibrosis that is provoked by continuous inflammation, vigorous cell proliferation, and excessive accumulation of the extracellular matrix (Tan et al., 2019). Clinically, keloids have individual tendencies, and they are liable to occur in exposed areas such as the sternum, scapula, and earlobe and are accompanied by pain and itching (Lee et al., 2004). The formation of postoperative keloids can have some negative and even devastating consequences for cosmetic surgery due to the lack of effective laboratory indicators to screen susceptible individuals. The current diagnosis of keloids relies heavily on clinical presentation, which is highly subjective and uncertain and does not provide prevention or early diagnosis (Berman et al., 2017). We aimed to discover diagnostic markers of keloids that could objectively assess the physiological status of patients and evaluate whether they have a tendency to develop keloids. This will facilitate early screening, diagnosis and prevention of keloid scars, and early control of keloid growth can lead to significant clinical outcomes.

This study identified TGM2 as a key molecule in keloids. Previous studies have suggested that keloids are closely associated with a sustained inflammatory response and infiltration of inflammatory factors and chemokines (Wang et al., 2020c). Accompanied by activation of inflammatory factors like interleukin six and infiltration of neutrophils, the perivascular dermis appears to have nodular proliferation of fibroblasts and excessive deposition of extracellular matrix (Potter et al., 2019; Wang et al., 2020c). The arrangement is intricately followed by the formation of a mass of rotating collagen fibers, which eventually leads to keloids (Andrews et al., 2016; Chen et al., 2019). This process is consistent with the results of the correlation analysis of TGM2 with extracellular matrix gene set and IL6 in this paper.

Considering that certain autoimmune diseases can result in tissue damage by initiating an inflammatory response, a long-term inflammatory response eventually leads to tissue fibrosis, such as skin fibrosis induced by scleroderma (Wynn and Ramalingam 2012; Sziksz et al., 2015; Sunda and Arowolo 2020). Therefore, we speculated that keloids might be related to immune infiltration and supplemented the comparison of keloids and normal tissues at the level of immune cells. The results of immune infiltration analysis indicated that the screened TGM2 was positively correlated with monocyte and natural killer cell. There were significant differences in the proportion of eight immune cells in keloid and non-keloid tissues, which was consistent with previous studies (Bagabir et al., 2012; Wang et al., 2021).

Currently, the exploration of biomarkers in the field of keloids is generally derived from speculation of similar diseases. By way of example, since keloids have to some extent tumor characteristics, it is speculated that some tumor biomarkers may also be exploited as keloid markers (Gál et al., 2017; Ud-Din and Bayat 2020). The genes that control apoptosis and growth in some tumors may regulate the occurrence of keloids. For example, the tumor necrosis factor-related apoptosis-inducing ligand (TRAIL), which is differentially expressed in a variety of malignant tumors, such as lymphoma, bladder cancer, melanoma, and esophageal cancer, is capable of exerting a selective apoptotic effect (Fulda 2014; Alexiou et al., 2015). It has been confirmed that knocking down the expression of TRAIL aggravates the fibrosis of lung tissue, and upregulating TRAIL causes skin fibrosis to be considerably inhibited (Braithwaite et al., 2018; Jiang et al., 2019). Mutations in the oncogene PTEN (phosphatase and tensin homolog deleted on chromosome ten) are detected in hematological tumors, brain gliomas, osteosarcoma and many other tumors and can be the target for microRNA-21–5p to reduce the migration of keloid fibroblasts and govern autophagy (Worby and Dixon 2014; Chen et al., 2018; Yan et al., 2020). Further, some scholars have also postulated key genes in keloids based on liver injury, muscle injury, scleroderma, cardiac hypertrophy, and other diseases with similar fibrotic processes. For example, fussel-15 has the ability to regulate the fibroblasts of keloids and scleroderma (Arndt et al., 2011); microRNA-29 influences the fibrosis process of various organs, such as the heart, liver, lung and the growth of keloids (Deng et al., 2017; Gallant-Behm et al., 2019; Xu et al., 2020). Although some crucial genes in keloid can be revealed according to the above-mentioned ideas, their screening efficiency is relatively low and not accurate enough. Based on sequencing data and bioinformatics methods, the identification of possible biomolecules for diseases including keloid is more accurate and reliable, and more efficient.

TGM2 belongs to the TGM protein family, which consists of structural and functional enzymes and participates in the posttranscriptional modification process of proteins by inducing cross-linking, transamination and deamination between proteins (Li et al., 2018; Lee et al., 2021). TGM2 participates in many pathological reactions, including inflammation, chemotaxis of inflammatory factors, tumor progression, wound healing, tissue fibrosis, and immune diseases (Cho et al., 2020). The members of this family exhibit a variety of enzyme activities, such as protein kinase, GTPase and ATPase activities, disulfide isomerase activity, and participate in cell adhesion.

Keloids are the consequence of a series of intertwined pathological processes consisting of excessive fibroblast proliferation, extracellular matrix deposition, and dysregulated modulation of cell migration. TGM2 has been reported to regulate cell cycle protein expression levels in the tumor microenvironment to aid cell cycle progression and facilitate fibroblast proliferation (Nadalutti et al., 2011; Lee et al., 2015). The transamidase activity of TGM2 contributes to the maturation and stability of collagen and extracellular matrix by cross-linking with extracellular matrix proteins and is involved in wound healing and fibrosis in various tissues, such as scar tissue, liver and heart (Wang et al., 2020a; Wang et al., 2020b). During collagen lattice formation, TGM2 plays a dominant role in the initial formation of calcium-dependent morphology and is involved in early extracellular matrix formation (Li et al., 2018). In many tumors with high metastatic potential, TGM2 expressed above basal levels is found to be associated with loss of cell polarity and disruption of cell junctions, which makes epithelial cells motile and invasive (Pierce et al., 2013; Eckert et al., 2014). The action process of TGM2 is closely connected with the various stages of tumor growth, including the epithelial-mesenchymal transition, apoptosis, differentiation, and aggressive metastatic phenotype formation of cancer stem cells. TGM2 regulates the angiogenesis of digestive system tumors through the WntB-catenin pathway and interferes with the apoptosis of colon cancer cells (Zhang and McCarty 2017; Torres et al., 2019). TGM2 is overexpressed in esophageal adenocarcinoma and attaches to tumor staging (Leicht et al., 2014). Although the study of TGM2 in keloid pathogenesis lacks in-depth research, it is reasonable to believe that TGM2 is a very promising mechanistic molecule in keloids based on its regulation of fibroblast and extracellular matrix in tumors.

This study finally identified TGM2 as a promising biomolecule in keloids. However, the limitations of this study must be acknowledged. On the one hand, this is a retrospective study of expression profiling data. It is undeniable that age, gender, and race are important influencing factors in the development of keloids. The correlation between keloid biomarkers and these factors still needs to be elucidated by large sample and multicenter studies, which is a very promising research direction. On the other hand, the analysis of immune infiltration is based on limited genetic data. Therefore, it may deviate from the interactions of immune cells in specific diseases, disease-induced disorders or the actual situations of individuals, and further molecular experiments are necessary for verification.

## Conclusion

TGM2 may be able to act as an auxiliary diagnostic indicator for keloids. TGM2 has been confirmed in previous studies to be associated with fibrosis and tumorigenesis; however, the role of TGM2 in keloids has not been adequately reported. Therefore, TGM2 may be developed as a new research hotspot and target for the prevention, diagnosis and treatment of keloids.

## Data Availability

The datasets presented in this study can be found in online repositories. The names of the repository/repositories and accession number(s) can be found in the article.
